# Exploration of factors affecting Australian students' mathematics grades: a multiple regression analysis based on PISA 2022 data

**DOI:** 10.3389/fpsyg.2025.1611350

**Published:** 2025-10-01

**Authors:** YuKai Wei, Yi Zhang

**Affiliations:** Faculty of Education, Beijing Normal University, Beijing, China

**Keywords:** math education, PISA, multiple regression analysis, secondary education, mathematics education in Australia

## Abstract

**Introduction:**

Currently, PISA (Programme for International Student Assessment) Mathematics Grades worldwide is declining, while Australia students' performance shows an upward trend. To promote mathematics education in Australia and share educational experiences, this study explores factors that impact Australia's PISA mathematics grades significantly, and identifies ways to improve mathematics performance. Guided by Bronfenbrenner's ecological systems theory, this study develops a dual-layer nested ecosystem model, including home context, information resources, personal feature, school environment, math teaching and learning.

**Methods:**

Data is from PISA 2022 datasets. The independent variables are also divided into five categories according to the theoretical framework. There are 33 variables and 6,386 pieces of data. This study uses SPSS to conduct multiple regression analysis. In this study, predictors are categorized into five models, adding one influencing factor type to each model one by one.

**Results:**

The factors in model 5 explain 51.1% of math grade changes. Home context has the strongest explanatory power, it explains 19.9% math grade changes. Home possession (β = 0.304) and ESCS (β = 0.266) benefit math performance. Math teaching and learning explains 17.2% of math grade changes. Mathematics self-efficacy: Formal and applied mathematics (MATHEFF) is more influential (β = 0.376).

**Discussion:**

This study provides meaningful implications for identifying key determinants of mathematics education outcomes, informing evidence-based policy refinement, and enhancing instructional practice design. The findings offer actionable insights for stakeholders seeking to optimize mathematics learning ecosystems. To improve math achievement, Math education resource equity and scientific math teaching content are important.

## 1 Introduction

### 1.1 Background

Math education has a profound impact on socioeconomic development. A high math development level is associated with more employment opportunities, higher wages, healthier physical and mental states, and lower crime rates. Increased mathematical ability is effective in increasing income and tax revenue and has positive implications for global economic growth and social development (Ramirez et al., 2018). Consequently, educational researchers have attempted to explore factors such as attitudes, motivation, and math teaching that influence mathematics achievement.

The Program for International Student Assessment (PISA) uses internationally recognized indicators to collect data from students, teachers, schools, and systems to understand differences in performance. PISA is designed to help schools and policymakers share data and move away from focusing on each other within the same education system to looking outward. PISA provides a reference for the education system and informs policy decisions in numerous countries. PISA emphasizes students' ability to apply knowledge creatively, think critically across disciplines, and demonstrate effective learning strategies (Schleicher, 2022).

Australia's education system is socioeconomically equitable, achieving inclusive education by achieving or exceeding Level 2 education. In 2022, it ranked 17th in math, reaching 487.08, which is above the OECD average score of 165. In Australia, 59.5% of 15-year-old students achieved or exceeded Level 2 in all three areas. Since 2018, both disadvantaged and advantaged students have improved their math performance. The data show that the isolation index for disadvantaged students is 0.2 and for advantaged students is 0.18. Both indices are below the OECD average. This implies that different types of pupils were more likely to be in contact with other pupils. Of the parents, 74% received regular information about their child's performance, and home-school links were strong. This figure is higher than the OECD's average. A total of 81% of the students said that their parents or family asked them at least once or twice a week what they did at school that day. Of these, 15% of boys achieve or are above Level 5 in mathematics, while 10% of girls achieve the same level (OECD, 2024b).

Changes in math performance were associated with social and emotional skills, with an 11.8 point difference in math performance for students with curiosity, a 10.3 point higher math performance for those with persistence, a 6.7 point higher performance in the Emotional Control group, a 5.9 point higher performance in the resilience group, a 4.1 point higher performance in the assertiveness group, and a 1.7 point lower performance in the cooperation group.

Australia's overall high performance in mathematics is a good reference for other countries to share their educational experiences and to promote the development of mathematics education. Different influencing factors may have different impacts in different countries and environments. Therefore, it is relevant and innovative to study the specific impact of influencing factors, especially the mathematics teaching process and mathematics teaching materials, in the Australian mathematics education environment.

Among Western nations, Australia has demonstrated particularly notable improvements in mathematics education outcomes, as reflected in its PISA rankings, distinguishing it from traditionally high-performing East Asian systems (e.g., China, Japan). This trajectory offers valuable insights for culturally analogous Western countries seeking to enhance their mathematics education frameworks.

Australia's PISA mathematics rankings over the past decade reveal a significant recovery in the most recent cycle: 19th (2012), 21st (2015), 29th (2018), and 16th (2022). This rebound—marking the first sustained improvement in recent years—underscores the importance of examining the drivers of this progress. Investigating the factors influencing Australian students' mathematics performance during this recovery phase may yield actionable strategies for reversing declining trends, particularly in countries struggling with mathematics education.

**Figure 1:**
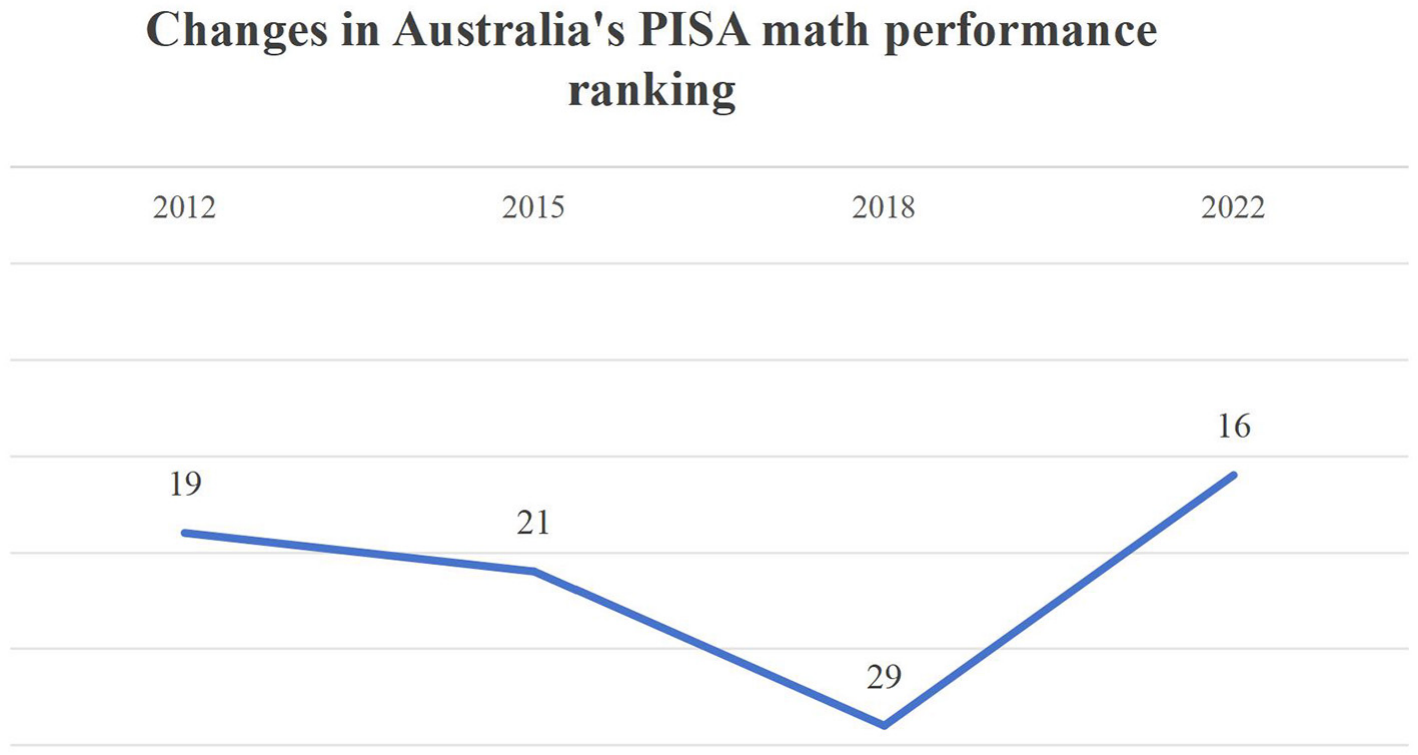
Changes in Australia's PISA math performance ranking.

Furthermore, Australia's mid-to-upper-performance tier enhances the transferability of its policies and practices. Unlike top-performing systems, whose exceptional contexts may limit the generalizability of their approaches, Australia's moderate yet upward-trending position makes its strategies more adaptable to countries aiming to achieve sustainable improvements in mathematics education quality (Figure 1).

This study adopts a hierarchical analysis approach using PISA 2022 questionnaire data to explore the factors that have the greatest influence on Australia's mathematics performance by categorizing the independent variables into five hierarchical dimensions: mathematics education materials, math learning and teaching process, students' characteristics, school context, and home environment.

### 1.2 Latest research developments

First, in the family model, researchers explored the role of parental involvement and home-math environments. Family serves as a critical context for early math learning, where parental attitudes, behaviors, and the quality of home math activities significantly shape children's mathematical development. Studies indicate that parents' math self-efficacy and the perceived value of math enhance their engagement, thereby improving children's math interest and performance through activities such as games and math-related conversations (Cheung et al., 2024). However, interventions show that merely fostering a growth mindset in parents (e.g., emphasizing math ability as malleable) does not necessarily translate into behavioral changes or improved child outcomes, suggesting complex mediating mechanisms (MacDonald et al., 2024). Additionally, maternal and paternal involvement differ; fathers often promote a growth mindset through play and challenging interactions, reducing math anxiety, while mothers tend to focus on structured learning (Cai et al., 2024). Socioeconomic status (SES) further influences home math environments—low-SES families face higher math anxiety, often relying on repetitive drills rather than integrating math into daily life, highlighting the need for culturally adaptive interventions and resource support (Lu et al., 2025).

Second, in the school model, the researchers examined the impact of instructional support and early intervention. School education plays a pivotal role in mitigating family disparities and in shaping mathematical learning trajectories. Students with early math difficulties (MD) can be categorized into three growth trajectories: severe (slow growth), moderate (steady improvement), and mild (near-typical levels), with cognitive factors (e.g., working memory and reading skills) and teacher assessments predicting their developmental paths. Personalized interventions, such as Individualized Education Programs (IEPs) or math tutoring, effectively address these difficulties (Gesuelli et al., 2025). Additionally, digital learning tools (e.g., adaptive math programs) reduce avoidance behaviors in anxious students, although overreliance on technology may neglect socio-emotional teacher-student interactions (Hilz et al., 2025). Thus, schools should integrate teachers' professional development (e.g., precise student needs assessment) with technology to foster inclusive math learning environments.

Third, in the individual model, the current research examined the interplay between cognitive abilities and affective factors. Children's cognitive skills (e.g., working memory and executive functions) and emotional experiences (e.g., math interest and anxiety) jointly shape their mathematical performance. Working memory partially mediates the relationship between math anxiety and achievement, as anxiety consumes cognitive resources and impairs problem-solving efficiency (Tapola et al., 2025). Distinct executive function components show differential effects; attentional focus directly and indirectly (via anxiety reduction) enhances math performance, whereas inhibitory control has weaker effects (Sánchez-Pérez et al., 2024). Additionally, early math interest (e.g., by Grade 3) predicts later advanced skill development (Kilp-Kabel and Mädamürk, 2024). Cultivating interest requires alignment with cognitive challenges—e.g., game-based learning can simultaneously improve statistical reasoning and arithmetic skills (DePascale and Ramani, 2024). However, most studies focus on isolated factors; future work should integrate cognitive, affective, and environmental models, such as investigating how family support buffers the negative impact of anxiety on working memory.

### 1.3 Theoretical framework

This study employed Bronfenbrenner's ecological systems theory as its theoretical framework, incorporating moderate adaptations to identify the most influential factors in different models affecting students' mathematics education outcomes. The theoretical framework is Bronfenbrenner's ecological theory of human development (Bronfenbrenner, 1981), which emphasizes the importance of systemic correlations and interactions between biological factors and the environment in human development (Wang et al., 2023). Academic achievement is not only influenced by the efforts and learning of teachers, schools, or individual students but is also associated with substantial interactions between various factors (Scheeren, 2022).

Bronfenbrenner's second-stage theory of human development is the person-process-context model, which categorizes factors into five dimensions. Based on the theory and current literature, this study modified the original theoretical model by designing five dimensions related to mathematics education: home context, information resources, personal features, school environment, math teaching, and learning.

Building on Bronfenbrenner's ecological theory of human development, this study proposes an innovative dual-layered nested ecosystem model to examine the factors influencing students' mathematics achievement, reflecting current trends in mathematics education research.

Bronfenbrenner's theory posits that individual development is shaped by nested multi-level environmental systems. Accordingly, Australian students' PISA mathematics performance can be analyzed through a dual-layered ecosystem: the out-of-school system (family, information resources, individual characteristics) and the in-school system (school environment, mathematics teaching, and learning). These layers interact dynamically to shape learning outcomes.

The first part focuses on the external school system. The outermost layer is the home context, in which family environments shape the foundational aspects of math learning. As the primary social unit, families influence math learning indirectly through socioeconomic status, parental educational beliefs, and home atmosphere. For instance, higher-income families may provide more learning resources (e.g., tutoring), while supportive environments reduce anxiety and stabilize the “foundation.”

The middle layer comprises the information resource system, bridging family empowerment and individual development. Community resources and online platforms serve as intermediaries. For example, family resources (home context) determine access to quality information (e.g., smart learning devices), which in turn creates learning opportunities (e.g., self-study via apps), fostering skill development.

The innermost layer comprises personal features, where individual traits create disparities in math ability. Under the combined influence of family and resources, students develop distinct cognitive skills and motivations. Children exposed to early math resources may develop stronger logical thinking, while those from math-neglectful homes may adopt a “math is useless” mindset.

The second part examines the in-school system directly impacting math performance. The fourth layer (outer school system) is the school environment, where resources and culture provide supportive conditions. Facilities, the classroom climate, and teacher quality form an external framework. Well-resourced schools (e.g., labs, competition coaching) can compensate for family disadvantages (external home context), creating a “protective effect.”

The fifth layer (inner school system) is math teaching and learning, where instructional practices directly determine the outcomes. Proximal factors such as teaching methods (e.g., inquiry-based vs. rote learning) shape engagement and anxiety levels and interact with personal features (e.g., interest or aversion).

Thus, the math education ecosystem follows an empowerment path from external to internal systems. Family (home context) shapes individual traits (personal features), influencing school adaptation, with information resources acting as a bridge. Meanwhile, school resources (school environment) and quality instruction (math teaching) further drive individual growth.

**Figure 2:**
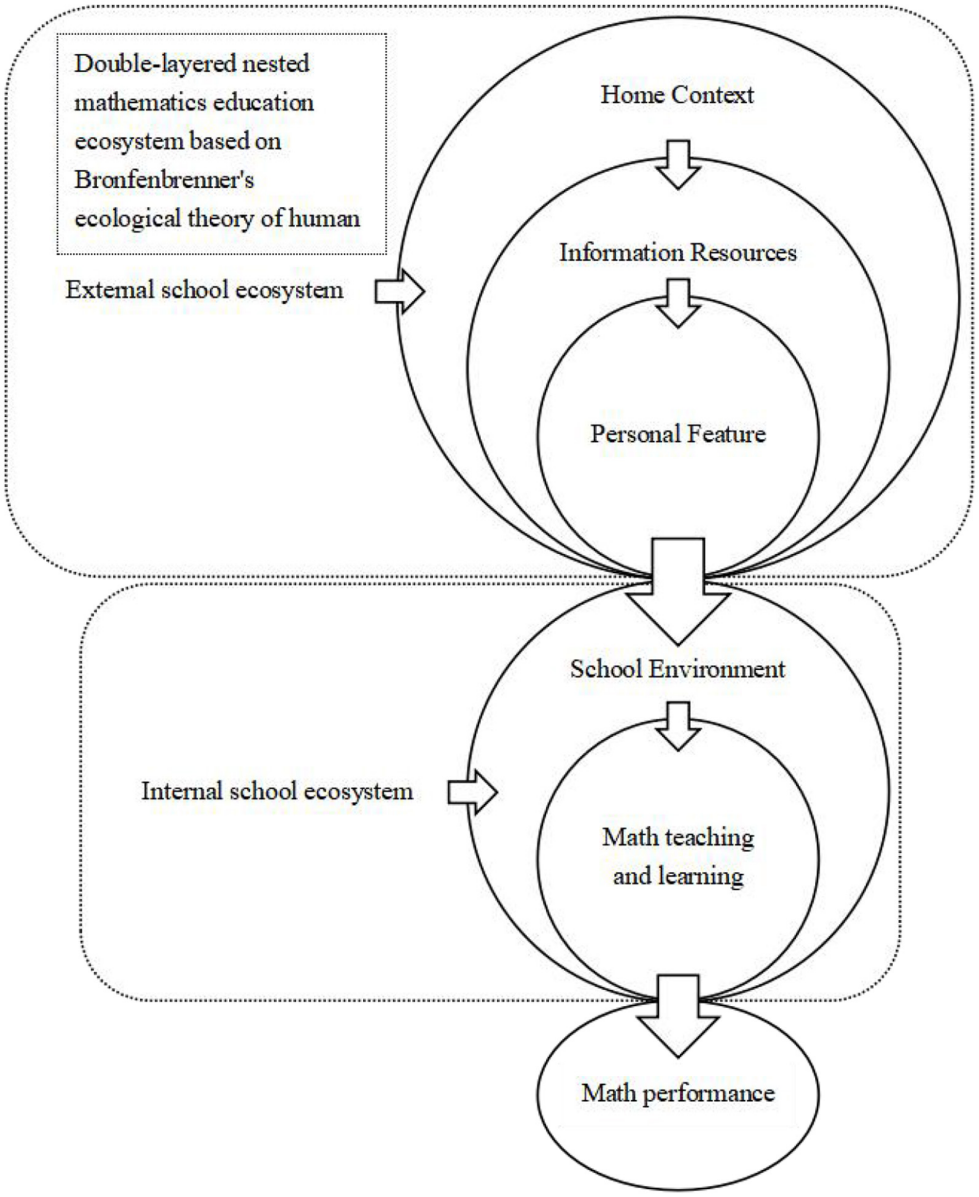
Conceptual diagram of the theoretical model.

In summary, math achievement results from the layered interactions of these factors across the dual systems (Figure 2).

### 1.4 Research gap and research questions

This study addresses research gaps and demonstrates innovation across three dimensions: the research object, content, and methodology.

First, in terms of the research object, this study examines PISA math education influencing factors in Australia, offering novel insights. Existing PISA math research primarily focuses on East Asian or Western developed nations, whereas systematic studies on Australia's math education system remain scarce. Despite Australia's stable and rising PISA math performance (ranked 16th in 2022), empirical studies of its success factors are limited. Australia's education system exhibits unique socioeconomic equity, with below-OECD average isolation indices for both disadvantaged and advantaged students. Yet, how this inclusive environment enhances math achievement remains underexplored. Compared to heavily researched PISA countries (e.g., the U.S. and China), Australia's significant progress in math—within a Western context—holds greater transferable value for culturally similar nations, although this potential remains understudied.

Second, in terms of research content innovation, this study breaks through the limitations of traditional mathematics education factor analysis by systematically incorporating specific mathematics teaching practice variables into the research framework for the first time. Although the current research is relatively comprehensive, there are few studies on math-related factors, such as teaching materials and the learning process. It is unclear which kind of teaching mode, class design, and math content are more influential. The specific role of math materials needs more exploration. This study focuses on teaching resources and modes that are directly related to mathematics education. Existing literature predominantly focuses on macro factors, such as student characteristics and family background, with insufficient attention to the mathematics teaching process itself, including instructional design, pedagogical approaches, and classroom practices. This study innovatively constructs a five-dimensional theoretical model, specifically isolating “mathematics teaching and learning” as an independent dimension to analyze core elements, such as teaching material quality and instructional effectiveness, that directly influence math achievement. This micro-level examination of teaching practices addresses a critical gap in prior research that overemphasizes external environmental factors while neglecting the intrinsic aspects of instruction.

Methodologically, this study employed Multiple Regression Analysis to systematically integrate five models of variables: family background, information resources, individual characteristics, school environment, and mathematics teaching. According to the logic of the theoretical framework, each model incrementally incorporates the independent variables associated with each layer of the framework. For example, Model 1 includes only home-text-related independent variables. Through this sequential addition, Model 5 encompasses all independent variables from the five dimensions. By stacking the models, this study investigates the factors with the greatest impact on mathematics education, with a particular focus on the role of mathematics teaching and learning-related variables.

The research questions are

What factors significantly influenced Australian students' PISA mathematics performance?

Which influencers directly related to mathematics teaching and learning have a more significant impact? Do the factors related to mathematics education and teaching have a significant impact on students' mathematics grades?

## 2 Literature review

The literature review is structured into two parts. First, we examined the factors influencing mathematics achievement, focusing on four key dimensions: individual student characteristics, family background, school environment, and the education system. The second was a review of relevant studies on mathematics education in Australia.

### 2.1 Factors affecting mathematics education

Factors influencing PISA math performance include individual student characteristics, family background, school environment, and education system (Wang et al., 2023).

#### 2.1.1 Demographic and psychological variables

Demographic variables affect math achievement. Males perform better in math (Gevrek et al., 2020). The frequency of using a test language at home is associated with math (Pivovarova and Powers, 2019a). Minority and immigrant status are associated with lower math scores, and immigrants perform worse than native students (Radl et al., 2017). Family background, such as economic status, structure, and school context, reduced the negative effects of ethnicity and immigrant status on math performance.

The higher the student grade level, the higher the mathematics performance (Spörlein and Schlueter, 2018). Students in higher grades are exposed to a wider range of math content that contributes to mathematics achievement (Barnard-Brak et al., 2018). Other studies indicate that the correlation between age and mathematics achievement is mixed, with some students demonstrating improved performance as they grow older, whereas others show declining results (Rodríguez et al., 2020).

Psychological factors affecting students' mathematics performance. Factors like student motivation, mathematical self-beliefs, self-efficacy, mathematical anxiety, mathematical self-concept, and mathematical dispositions all have an impact on math performance.

Positive math self-efficacy provides a strong driver for performance. The stronger the internal drive and motivation to learn mathematics, the higher their mathematics achievement (Bhutoria and Aljabri, 2022). Mathematics self-efficacy has a positive effect on mathematics achievement (Zhao and Ding, 2019). Expectations of success and value in math tasks influence math learning choices and performance (Schukajlow et al., 2023). These expectations are similar to concepts such as self-concept and self-efficacy.

Mathematical anxiety is negatively correlated with math performance (Zhao and Ding, 2019). Math anxiety is an emotional tendency to react anxiously to math exam situations. It triggers fear of failure, nervousness, and worry (Schukajlow et al., 2023). Math self-concept and intention have mixed effects (Guglielmi and Brekke, 2018; Kitsantas et al., 2020). The relationship between subjective norms and mathematical achievement is complex. Higher subjective norms promote math achievement by closing the gender gap (Munir and Winter-Ebmer, 2018).

Furthermore, in mathematics education, mindfulness and psychological wellbeing help to mitigate students' math anxiety and school refusal tendencies. Research demonstrates that mindfulness training enhances students' focus during mathematical learning and reduces negative emotions (e.g., anxiety and avoidance) stemming from poor performance. Self-awareness aids students in recognizing their emotional responses to math learning, whereas emotional regulation alleviates task-induced stress and boosts confidence. By cultivating these psychological skills, students develop a more positive attitude toward mathematical challenges, reducing anxiety-driven avoidance and thereby improving learning satisfaction and engagement (Sorrenti et al., 2024, 2025). Thus, integrating mindfulness practice into math instruction can enhance psychosocial adaptability and foster academic commitment.

In summary, existing studies have systematically examined the relationships between demographic factors, psychological variables, and math achievement. However, the mechanisms underlying gender, immigrant status, and other variables remain unclear, necessitating deeper sociocultural analysis. Psychological variables interact dynamically (e.g., self-efficacy may buffer the negative effects of math anxiety), warranting further longitudinal investigations. The “mixed correlation” between grade and achievement suggests age is not deterministic, requiring instructional interventions as moderators to explain variations. Future research should integrate multi-level variables and develop non-linear models to better elucidate the mechanisms influencing math performance.

#### 2.1.2 Family background

The household context has an impact on math achievement. There was a positive correlation between the two-parent families and math achievement. Students living in extended households have lower achievement levels (Yamamura, 2019). The effect of family SES (family economic status) was positively correlated with math achievement. SES (socioeconomic status) indirectly influences students' mathematics achievement through educational resources, such as the family environment and sociocultural capital (Driessen et al., 2005). There is a complex link between family SES as an indicator of SES and TIMSS math achievement. Home possessions, educational resources, books available at home, and parental educational background tend to be positively associated with math performance. The presence of books, Internet access, and the learning environment contribute to the better academic performance of students (Younes et al., 2023). Household wealth, family structure, and cultural wealth have mixed effects (Tan and Hew, 2019; Xie and Ma, 2019). Most studies have found a negative or insignificant association between household ICT availability and math achievement (Tan and Hew, 2019).

In most countries, cultural appropriation positively mediates the relationship between parents' occupational status, family wealth, and mathematical achievements. Parental expectations and behaviors include many sub-factors that have a positive influence, such as parental higher education and occupational and academic expectations (Tan, 2017). Parental education is positively related to math achievement (Gimenez et al., 2018). Parents' education duration has a strong predictive power (Giannelli and Rapallini, 2019). Fathers' education levels are more important than those of mothers (Dockery et al., 2020), while mothers' education levels reflect the type of support they provide for their children (Younes et al., 2023). Parents' occupational status had mixed effects. Fathers' occupational status is positively associated with math achievement, and mothers' occupational status may be more influential (Erdogdu, 2022). Fathers' part-time jobs are negatively associated with math achievement, whereas mothers' part-time jobs are positively associated with math achievement (Giannelli and Rapallini, 2016). Supportive parental behavior, including emotional support, also has an impact on achievement.

The reviewed studies demonstrated that family background influences mathematics achievement in both multidimensional and non-linear ways. While two-parent households, higher SES, and educational resources (e.g., books and Internet access) generally correlate positively with achievement, the effects of family structure (e.g., extended families) and ICT availability remain contested—suggesting that the conversion of socioeconomic resources into educational advantages is mediated by cultural and familial dynamics. The differential impacts of parental education and occupational status (e.g., fathers' education being more crucial and mothers' employment showing stronger effects) reflect gendered parenting roles. Moreover, the mixed associations of certain variables (e.g., parents' part-time work) highlight the complex trade-offs between family economic strategies and educational investment. Future research should further disentangle the interplay of family capital (economic, cultural, and social) across cultural contexts to avoid oversimplified causal interpretations.

#### 2.1.3 School environment

The school environment affects students' math performance. Schools with high SES outperform those with low SES in terms of mathematical literacy. The school environment includes classroom and teacher characteristics, pedagogy, and assessment (Wang et al., 2023).

Student learning is related to math achievement. Learning opportunities have an impact on math achievement (Ünal et al., 2022). Moderate use of ICT is associated with higher mathematics achievement (Bhutoria and Aljabri, 2022). Students' behavior and engagement in school have an impact on mathematics achievement. Generally, positive attitudes toward school are positively associated with mathematics achievement (King et al., 2020), and there is a negative correlation between absenteeism, tardiness, and achievement (Yamamura, 2019). The relationship between the incidence of student misbehavior and achievement has been negatively correlated. This includes the prevalence of bullying in schools, the frequency of students being disrupted (Spörlein and Schlueter, 2018), and overall student misbehavior (Pivovarova and Powers, 2019b).

Moreover, the relationship between students' learning approaches and mathematics achievement depended on the type of learning approach. Metacognitive strategies are positively related to mathematics achievement. Memory, competitive learning, and cooperative learning are negatively related to mathematics achievement (Bhutoria and Aljabri, 2022).

Students' perceived school climate indirectly affects mathematics achievement through school refusal behaviors. A sense of school belonging reduces difficult transitions, thereby enhancing learning engagement, whereas rigid educational environments exacerbate anxious anticipation and interpersonal discomfort, leading to increased absenteeism. These school refusal behaviors directly diminish academic involvement and contribute to underachievement, ultimately affecting performance in mathematics and other subjects. Classroom characteristics consist of disciplinary climate, class size, teacher-student ratio, and regular class time. The results of the relationship between disciplinary climate and math achievement have been mixed (Cheema and Kitsantas, 2014). In data from four PISA cycles from multiple countries, there was a positive correlation between disciplinary climate and achievement (Caro et al., 2016). There was no negative correlation between the classroom climate and math achievement. Regular class time tends to be positively correlated with math achievement (Santibañez and Fagioli, 2016).

Teacher characteristics comprise teacher affective quality (usually measured in terms of teacher-student relationships) and teacher qualifications. Teachers' affective quality is positively correlated with achievement, and there is a weak negative association (Lee, 2016, 2021). Research has found that the relationship between teacher-student relationships and mathematics is positive in student and school environments in most countries, with mixed results for the association between teachers' professional development and math achievement (Caro et al., 2016). In a supportive classroom environment, positive teacher-student relationships are associated with higher levels of student motivation, favoring the development of student expectations and values (Durksen et al., 2017). Teaching methods, assessments, and content coverage affect math achievement. The results regarding the relationship between cognitive activation and mathematics learning are mixed. It is related to the country's economic level, students' ethnicity, and type and frequency of cognitive activation activities (Santibañez and Fagioli, 2016).

There are both positive and negative correlations between IT use in schools and math achievement (Skryabin et al., 2015; Erdogdu, 2022). ICT infrastructure has a positive effect on math achievement (Chiu, 2020). The ratio of ICT to school size and the effect of ICT shortages were not significant (Tan and Hew, 2019). More computers per student hinder math achievement (Sousa et al., 2012).

In summary, school environments influence mathematics achievement through complex context-dependent mechanisms. While high-SES schools generally demonstrate better performance, the effects of specific factors (e.g., classroom discipline and teacher-student relationships) vary across countries and economic levels, highlighting the crucial interaction between structural resources and teaching practices. The impact of ICT appears particularly paradoxical: moderate use enhances learning, whereas excessive provision (e.g., high computer-to-student ratios) may hinder achievement, underscoring the need for alignment between technological tools and pedagogical strategies. Furthermore, inconsistent effects of teacher emotional support vs. cognitive activation suggest that instructional effectiveness depends not merely on qualifications but more critically on teacher-student interaction quality and classroom culture. Future research should better delineate how school environment variables operate across different educational systems to avoid oversimplified attributions to single factors.

#### 2.1.4 Macro educational system

Factors such as school composition, type, location, resources, and size affect students' performance in mathematics. Repetition rates, dropout rates, teacher and staff shortages, and the incidence of school misbehavior are significantly negatively correlated with mathematics achievement. School socioeconomic status composition, general academic school, and educational resources were positively correlated. Financial resources for education, decentralization, and tracking are three main indicators in the education system. The related results are mixed. Financial resources for education report mixed results regarding the relationship between financial resources for education and mathematics achievement. There is a positive correlation between poor- and middle-income countries, but the returns diminish or disappear in high-expenditure countries (Breton, 2021).

Socioeconomic factors include gender equality and economic development, which are associated with mixed results (Schmidt et al., 2015). Different results vary depending on the level of economic development of the country, the PISA cycle, and gender equality measures (Anghel et al., 2020; Eriksson et al., 2020).

Information and communication technology (ICT) influences math performance (Courtney et al., 2022). Controlling for demographics, ICT availability at home significantly and negatively predicted student achievement in math and science, whereas no significant contribution was found on the school side. ICT use and attitude variables significantly predict students' achievement in math and science. Sustained engagement with ICT tends to be associated with lower achievement; therefore, ICT usage requires scientific planning (Ünal et al., 2022). When teachers integrate ICT into the classroom, students become more actively engaged in learning. Basic and advanced technology skills mediate the relationship between instructional and traditional technology use, which improves academic performance (Afari et al., 2023). One study investigated the importance of ICT use for the math achievement of Italian secondary school students, with a special focus on the mediating role of gender. There was a positive association between ICT use and math achievement when computers were used for specific activities. In the other cases, the correlation was negative. Girls' ICT is weakly associated with their math performance (Meggiolaro, 2017).

The following factors positively influenced mathematics education: grade level, age, and mathematics self-efficacy from the individual student model; overall family self-efficacy, overall family property, family educational resources, and parental educational background from the family context; and composition of school self-efficacy, general academic school, school educational resources, teacher credentials, regular school hours, and content coverage from the school context had a positive influence. Grade repetition, school dropout rates, student absenteeism, the incidence of student misbehavior, math anxiety, teacher and staff shortages, and student-centered instruction in the school environment model have a negative impact (Wang et al., 2023).

This review reveals complex yet inconsistent relationships between macro-educational system factors and students' mathematics achievement. For instance, while education expenditure shows positive correlations with math performance in low-income countries, its effects diminish in high-spending nations, suggesting that the marginal returns of resource investment are constrained by development levels and require a differentiated analysis based on national economic stages.

### 2.2 Current status of mathematics education in Australia

In Australia, motivation promotes math achievement (Gabriel et al., 2020). Speaking English as a Second Language decreases immigrant math proficiency (Dockery et al., 2020). In Australia, older students performed worse than younger students. Internet connectivity has a positive influence in Australia (Eickelmann et al., 2017). Family wealth was negatively correlated with math performance in Australia.

Approach, achievement, classroom goal structure, perceived peer valuing of mathematics, and teacher enthusiasm differentially predicted math performance (Beswick et al., 2022). Positive teacher-student relationships foster students' expectations and values, as teachers are sources of positive social influence (Wigfield and Gladstone, 2019). Positive teacher-student interactions in a supportive classroom environment promote student motivation and engagement (Durksen et al., 2017). There was also a correlation between math anxiety and math achievement. Mathematics test anxiety is a temporary, stable, and habitual emotional disposition that responds to test situations in mathematics. It triggers fear of failure, nervousness, worry, and physiological arousal (Schukajlow et al., 2023). Therefore, the overall effects of anxiety on task performance were negative for most students and under most conditions. The relationship between math anxiety and test scores is negative (Namkung et al., 2019; Zhang et al., 2019; Barroso et al., 2021).

The current state of mathematics education in Australia includes multidimensional influencing factors. External elements, such as motivation, language background, age, and Internet access, show complex associations with mathematics achievement, while internal classroom factors, such as instructional structure, peer assessment, and teacher-student interactions, affect learning outcomes through socio-emotional mechanisms. Notably, studies reveal a counterintuitive negative correlation between family wealth and math performance, warranting a deeper investigation into potential cultural or institutional explanations. Research on mathematics anxiety proves particularly insightful, not only confirming the emotion's direct interference with cognition but also highlighting the significance of non-cognitive factors in education. However, existing studies predominantly establish correlations without examining causal mechanisms or sufficiently accounting for the interaction effects among variables. Future research should employ longitudinal designs to distinguish between situational and trait anxiety effects while exploring the efficacy of targeted interventions.

## 3 Method and data

### 3.1 Method

This study employed the Statistical Package for the Social Sciences (SPSS) as analytical software. SPSS is a statistical analysis package designed for data processing, analysis, and visualization, offering comprehensive tools for researchers to extract information, support decision-making, and identify patterns. The specific version used was SPSS version 26.0.

The analysis used multiple regression analysis. Multiple regression analysis is a statistical method that is used to examine the linear relationship between a dependent variable and multiple independent variables. It extends simple linear regression (which involves only one independent variable) by simultaneously assessing the influence of multiple factors on the outcome variable while controlling for the effects of other variables. The formula for the research method is as follows:


$$\begin{array}{l} Y=\beta_{0}+\beta_{1}^{*}X_{1}+\beta_{2}^{*}X_{2}+......+\beta_{k}^{*}X_{k}+\varepsilon \end{array}$$

where *Y* is the value of the dependent variable, β_0_...β_6_is the regression coefficient of the model, which represents the effect of the independent variable on the dependent variable, *X*_1_...*X*_6_ are the values of the independent variable, and ε is the error term, which represents the factors that the model cannot explain. *p* < 0.05 indicates that the model is meaningful, and the *R*^2^ stands for the degree of the model's fit.

This study first processed the data and then conducted the basic reliability tests. Building upon an innovative adaptation of Bronfenbrenner's ecological systems theory of human development, this study designed five models to implement the sequential addition of variables in multiple regressions across different models. Because this research is based on theoretical nesting rather than the nesting of real-world data structures, it employs multiple regression models rather than Hierarchical Linear Modeling (HLM). The incremental addition of variables in multiple regression allows for controlling for confounding variables, enhancing model explanatory power (*R*^2^), addressing omitted variable bias, and facilitating model comparison and selection. If newly added variables significantly improve the predictive accuracy (e.g., by increasing *R*^2^), they are considered important independent variables. This approach enabled the study to specifically examine the role of school mathematics learning environments and mathematics instruction-related variables.

With the IRT, the dependent variables composed of educational items in the Main Survey Version questionnaire results were officially offered by PISA 2022. CFA is used to ensure that these manually stratified and averaged data can indeed be categorized into different models, and thus each factor is a stratification, which is quantitatively scientific. The final PV value of the dependent variable (explanatory variable) is a composite score obtained using principal component analysis (ETS Core A, 2021; OECD, 2024a,c).

### 3.2 Data

The data were extracted from the OECD website. After data cleaning, 6,386 pieces of data were processed. According to the classification based on a literature review and theoretical frameworks, as well as the availability of official data, the specific variables are as follows (OECD, 2024a). Home context includes Family support (FAMSUP), Family support for self-directed learning (FAMSUPSL), ICT resources (Information and Communication Technology resources, ICTRES), Home possessions (HOMEPOS), and the Complex composite index—Index of economic, social, and cultural status (ESCS).

Information resources included ICT availability at school (ICTSCH), Quality of access to ICT (ICTQUAL), Subject-related ICT use during lessons (ICTSUBJ), Use of ICT in inquiry-based learning activities (ICTENQ), and Support or feedback via ICT (ICTFEED).

Personal features include Sense of belonging (BELONG), Being bullied (BULLIED), Feeling safe (FEELSAFE), Perseverance (PERSEVAGR), Curiosity (CURIOAGR), Cooperation (COOPAGR), Empathy (EMPATAGR), Assertiveness (ASSERAGR), Stress resistance (STRESAGR), and Emotional control (EMOCOAGR).

The school environment includes the quality of student-teacher relationships (RELATST), school actions to sustain learning (SCHSUST), types of learning resources used while the school was closed (LEARRES), problems with self-directed learning (PROBSELF), feelings about learning at home (FEELLAH), and self-directed learning self-efficacy (SDLEFF).

Math teaching and learning includes Disciplinary climate in mathematics (DISCLIM), Mathematics teacher support (TEACHSUP), Cognitive activation in mathematics: Foster reasoning (COGACRCO), Cognitive activation in mathematics: Encourage mathematical thinking (COGACMCO), Exposure to formal and applied mathematics tasks (EXPOFA), Exposure to mathematical reasoning and twenty-first century mathematics tasks (EXPO21ST), Mathematics self-efficacy: Formal and applied mathematics (MATHEFF), Mathematics self-efficacy: Mathematical reasoning and twenty-first century mathematics (MATHEF21), Subjective familiarity with mathematics concepts (FAMCON), Mathematics anxiety (ANXMAT), and Proactive mathematics study behavior (MATHPERS).

PISA 2022 uses the Item Response Theory (IRT) approach in the analysis, data scaling, and measurement of trends across cycles. The IRT model implemented a hybrid model that combined a Rasch approach with a two-parameter logistic model and a generalized partial credit model (GPCM) to increase the scaling ability and address the complexities of PISA response data. The specific educational items, attitudes, and fields in the questionnaire were integrated as related influencing factors. Then, with CFA, the influencing factors, as secondary indicators, were synthesized into different models.

## 4 Results

### 4.1 Reliability and validity test

The alpha coefficient was 0.74, which indicates that the data reliability was high. Sample size is 6,386. The KMO value is 0.855, which is higher than 0.8, indicating that the data are suitable for information extraction. Therefore, the processed data had a high reliability and validity.

### 4.2 Confirmatory factor analysis

Guided by the literature review and established theoretical framework, this study employed confirmatory factor analysis (CFA) to examine the validity of the five-model structure. The CFA approach was adopted because the preliminary classification of factors influencing mathematics education was already in Bronfenbrenner's ecological theory of human development, thus allowing for empirical verification within this conceptual framework. Confirmatory factor analysis was conducted for the hierarchical situation. This Confirmatory factor analysis (CFA) analysis is carried out for a total of five factors and 37 analysis items. The effective sample size of this analysis is 6,386, which is 10 times more than the number of analyzed items, and the sample size is moderate (Table 1).

**Table 1:** Basic summary of CFA analysis.

**Factor**	**Number**
Home context	5
Information resources	5
Personal feature	10
School environment	6
Math teaching and learning	11
Account	37
Analysis sample size	6,386

Factor loading coefficient (FLC) values show the correlation between the factors (latent variables) and analytic items (explicit variables and measurement items). The standardized loading coefficient value is typically used to indicate the correlation between the factor and analytic term (measured term). If an item is significant, it indicates strong correlation. In this table, all items are significant (*p* < 0.05), and most of the standardized loading coefficients are higher than 0.4, indicating a correlation between the factors and the analyzed items (measured items; Table 2).

**Table 2:** Factor loading coefficient table.

**Factor (latent variable)**	**Measurement item (explicit variable)**	**Non-standard load factor (Coef.)**	**Std. Error**	***z* (CR)**	***p***	**Std. Estimate**	**SMC**
Home context	FAMSUP	1.000	–	–	–	0.156	0.024
	FAMSUPSL	0.782	0.107	7.300	0.000	0.111	0.012
	ICTRES	4.369	0.349	12.502	0.000	0.720	0.518
	HOMEPOS	5.881	0.469	12.535	0.000	1.000	1.000
	ESCS	4.177	0.334	12.493	0.000	0.709	0.503
Information resources	ICTSCH	1.000	–	–	–	0.012	0.000
	ICTQUAL	−58.188	1.393	−41.786	0.000	−0.480	0.230
	ICTSUBJ	−47.212	1.212	−38.968	0.000	−0.493	0.243
	ICTENQ	−71.514	1.151	−62.140	0.000	−0.692	0.479
	ICTFEED	−67.279	1.227	−54.816	0.000	−0.595	0.354
Personal feature	BELONG	1.000	–	–	–	0.601	0.361
	EMOCOAGR	0.962	0.030	31.652	0.000	0.502	0.252
	BULLIED	−0.587	0.029	−19.961	0.000	−0.294	0.086
	FEELSAFE	0.865	0.029	30.038	0.000	0.470	0.221
	PERSEVAGR	1.073	0.029	36.570	0.000	0.612	0.374
	CURIOAGR	0.881	0.029	30.311	0.000	0.475	0.226
	COOPAGR	0.807	0.027	30.454	0.000	0.478	0.229
	EMPATAGR	0.603	0.026	22.769	0.000	0.340	0.115
	ASSERAGR	0.842	0.031	27.307	0.000	0.419	0.175
	STRESAGR	0.828	0.029	28.737	0.000	0.445	0.198
School environment	RELATST	1.000	–	–	–	0.464	0.216
	SCHSUST	1.074	0.041	25.941	0.000	0.477	0.227
	LEARRES	1.089	0.042	26.079	0.000	0.481	0.232
	PROBSELF	−0.344	0.032	−10.715	0.000	−0.158	0.025
	FEELLAH	1.334	0.046	29.272	0.000	0.599	0.359
	SDLEFF	1.552	0.050	30.937	0.000	0.687	0.472
Math teaching and learning	DISCLIM	1.000	–	–	–	0.276	0.076
	ANXMAT	−1.784	0.099	−17.952	0.000	−0.417	0.174
	MATHPERS	2.016	0.104	19.392	0.000	0.548	0.301
	TEACHSUP	1.649	0.095	17.384	0.000	0.381	0.145
	COGACRCO	1.303	0.078	16.737	0.000	0.347	0.120
	COGACMCO	1.542	0.087	17.815	0.000	0.407	0.166
	EXPOFA	1.216	0.072	16.956	0.000	0.358	0.128
	EXPO21ST	1.533	0.085	18.101	0.000	0.427	0.182
	MATHEFF	3.551	0.172	20.591	0.000	0.779	0.607
	MATHEF21	2.901	0.141	20.614	0.000	0.787	0.619
	FAMCON	3.402	0.171	19.889	0.000	0.620	0.384

The horizontal bar ‘–' indicates that the item is a reference item.

The model fitting indicators are as follows. There are several fit indices. It is difficult for all indicators to meet the standard as long as a portion of the indicators that meet the standard is sufficient. In this study, RMSEA = 0.073, PGFI = 0.717, PNFI = 0.619, PCFI = 0.625, and SRMR = 0.078. A root mean square error of approximation (RMSEA) < 0.1 or < 0.08 may be acceptable. Standardized RMR (SRMR) < 0.1 may be acceptable (Doll et al., 1994). In existing research, PGFI/PNFI/PCFI ≥ 0.50 serves as the minimum acceptable threshold for models and represents the most fundamental baseline. When all three indices meet or exceed 0.50, the model demonstrates the basic explanatory power. These findings indicate that this study satisfies the standards for acceptable fit (Abd-El-Fattah, 2010; de la Rubia et al., 2020; Sathyanarayana and Mohanasundaram, 2024; Syah et al., 2025). Therefore, overall, the goodness-of-fit of this study meets the basic requirements, and the model fitting is acceptable (Table 3).

**Table 3:** Model fitting indicators.

**Common indicators**	**RMSEA**	**PGFI**	**PNFI**	**PCFI**	**SRMR**
Judgment criteria	< 0.10	>0.5	>0.5	>0.5	< 0.1
Value	0.073	0.717	0.619	0.625	0.078

### 4.3 Multiple regression analysis results

This study was divided into five models: Home context, Information resources, Personal feature, School environment, and Math teaching and learning.

#### 4.3.1 Home context model

The family model explores how students' family educational capital influences math performance.

Family support (β = 0.033), home possessions (β = 0.304), and economic, social, and cultural status (β = 0.266) positively affected math performance. Parents who discuss how well students are doing at school, eat meals with them, talk with them about problems, future education, and relationships, encourage them, take an interest in what they are learning, and ask what they did in school may help students achieve higher grades. The ESCS score is based on three indicators: highest parental occupation status (HISEI), highest education of parents in years (PAREDINT), and home possessions (HOMEPOS). The rationale for using these three components, which are consistent with the components used in previous PISA cycles, is that socioeconomic status is most commonly theoretically conceptualized based on “the big 3” (occupational status, education, and income). Parents with high occupational status and years of education were more likely to improve students' math performance. Students with more digital devices, books, educational software, and other materials, such as cultural capital and rooms, are generally better at math.

Family support, self-learning, and information resources negatively affected math education. Family support for self-directed learning, including help with homework, creating a learning schedule, access to learning materials online, finding additional learning resources, explaining new content, and checking school assignments, all resulted in lower math grades. Information and Communications Technologies (ICT) resources hinder math learning. Various digital devices and Internet access may distract students from learning math.

Family support and material resources (e.g., digital devices and books) positively influence mathematics achievement, consistent with current findings that high-SES families more effectively integrate math into daily life through informal activities, such as games (Lu et al., 2025). The quality of parental math talk (particularly unstructured interactions) most significantly promotes 3–5-year-olds' mathematical development (Silver et al., 2024). However, family directed autonomous learning shows negative effects, likely because excessive intervention undermines student independence, potentially due to parents' math anxiety, leading to overstructured tutoring (MacDonald et al., 2024).

#### 4.3.2 Information resources model

Regarding information resources, quality of access to ICT (ICTQUAL; β = 0.167) and subject-related ICT use during lessons (ICTSUBJ; β = 0.067) had a significant positive effect on math achievement. Sufficient digital resources, sufficient Internet speed, digital resources with proper function and easy access, technical support from schools and teachers with the necessary skills, and willingness to use digital devices during instruction all benefit math learning. Frequent use of digital resources in lessons in test languages, Mathematics, Science, and Computer Science promotes math performance.

Support or feedback via ICT negatively affected math performance, *B* = −0.349. Feedback sent by teachers and students regarding work and academic results, and work on drill and practice exercises using educational software or apps, hinders math learning.

The information resources model demonstrates that ICT quality and subject-specific usage positively affect mathematics achievement, with technological interventions (e.g., visualization software) effectively enhancing mathematical self-efficacy (Granello et al., 2025). However, ICT feedback support may yield negative effects, as real-time corrections could exacerbate anxiety and math anxiety, indirectly impairing performance by depleting working memory resources (Tapola et al., 2025).

#### 4.3.3 Personal feature model

The third model comprises personal features.

In terms of personal features, students who Feel Safe (FEELSAFE) perform well in math, β = 0.113. Students who feel safe in school, classrooms, and other places, as well as on their way to school and home, may have higher math grades.

Students with Resilient Perseverance (PERSEVAGR) perform well in math, β = 0.094. Students who work on a task until it is finished, apply additional effort when work becomes challenging, finish tasks that become boring, and are more persistent usually get higher math grades.

Students with Curiosity (CURIOAGR) perform well in math, β = 0.172. Students who are curious about many different things, such as asking questions, like to know how things work, love learning new things in school, like developing hypotheses, and check them based on observation, can perform better in math.

Students with Assertiveness (ASSERAGR) perform well in math, β = 0.032. Students who are comfortable taking the lead role in a group, know how to convince others, enjoy leading others, and keep their opinions to themselves in group discussions get higher math grades.

Students with Emotional Control (EMOCOAGR) perform well in math, β = 0.074. Students who keep their emotions under control, stay calm even in tense situations, and know how to control their feelings are better at math.

A high sense of Belongingness (BELONG) decreases students' performance in math, β = −0.035. Students who make friends easily at school and feel like they belong to school may perform poorly in math.

Being Bullied (BULLIED) can also cause students to do worse in math, β = −0.074. The typical manifestations of being bullied are being made fun of, being threatened by other students, being fought, being blackmailed, and having personal property destroyed.

Cooperation (COOPAGR) may make students perform worse in math, β = −0.078. In the basic education stage, students' cooperation to solve problems may lead to a lack of independent thinking and research abilities, which is not conducive to improving math scores.

The personal feature model reveals that non-cognitive factors, such as curiosity and emotional regulation, positively influence outcomes, while higher-order cognitive abilities (e.g., reasoning) enhance mathematical performance by integrating working memory resources (Esposito et al., 2025). The detrimental effects of bullying are confirmed, as victimization reduces effort and self-control, increasing vulnerability to anxiety and lowering math achievement (Sánchez-Pérez et al., 2024). However, strong peer belonging may foster over-reliance on social support, potentially limiting independent thinking and resulting in weaker math skills (Gesuelli et al., 2025).

#### 4.3.4 School environment model

The school environment was the fourth model.

High-quality Student-Teacher Relationships (RELATST) increase students' performance in math, β = 0.083. When teachers are respectful toward, concerned about, and friendly to students and are interested in their wellbeing, students can perform better in math.

School actions to Sustain Learning (SCHSUST) promotes achievement in math, β = 0.095. When schools send students learning materials to study on their own, send assignments and helpful tips, upload material on a learning management system or school learning platform, offer live virtual classes on a video communication program, and check in with students to ensure that they are completing assignments, students can often get better math performance.

Self-Directed Learning Self-Efficacy (SDLEFF) promotes achievement in mathematics, β = 0.071. Students who can use a learning management system or school learning platform, a video communication program, find learning resources online on their own, motivate themselves, complete school work independently, and assess progress with learning can perform better in math.

Problems with self-directed learning (PROBSELF) can lead to lower math scores, β = −0.049. Problems with access to digital devices, the Internet, school supplies, a quiet place to study, and problems understanding school assignments and academic assistance all lower math grades.

The school environment model indicates that teacher-student relationship quality and school-provided autonomous learning support create an adaptive learning environment conducive to mathematics achievement, effectively reducing avoidance behaviors among anxious students and enhancing math performance (Hilz et al., 2025).

#### 4.3.5 Math teaching and learning model

The fifth model concerns math teaching and learning. Disciplinary climate in mathematics (DISCLIM) leads to positive achievements in mathematics, β = 0.070. Learning venues without noise and disorder that help students focus and work well can promote math learning. A classroom with a better learning atmosphere prevents students from being distracted by digital resources.

Mathematics teacher support (TEACHSUP) leads to higher math scores, β = 0.025. When teachers show an interest in every student's learning, give extra help with math learning when they need it, and continue teaching until the students understand, they can perform better in math.

Cognitive Activation in mathematics foster reasoning (COGACRCO) had a positive influence on math achievement, β = 0.022. Teachers' practices of cognitive activation in math include asking students to solve mathematics problems without computing anything, to explain how students solved a mathematics problem, to explain what assumptions students were making when solving a mathematics problem, to explain students' reasoning when solving a mathematics problem, to defend students' answers to a mathematics problem, and to think about how new and old mathematics topics were related, encourage students to think about how to solve mathematics problems in different ways than demonstrated in class, tell students to keep trying even when facing difficulties with a mathematics task, and memorize rules and apply them to solve mathematics problems.

Mathematics self-efficacy: Formal and applied mathematics (MATHEFF) helps students perform well in math, β = 0.376. MATHEFF includes train timetable questions, calculating how much more expensive a computer would be after adding tax, calculating how many square meters of tiles you need to cover a floor, understanding the scientific tables presented in an article, solving various equations, finding the actual distance between two places on a map, and calculating the power consumption of an electronic appliance per week. Students who are confident in those tasks perform better in math.

Exposure to mathematical reasoning and twenty-first-century mathematics tasks (EXPO21ST) can lead to higher math scores, β = 0.025. EXPO21ST includes extracting mathematical information from diagrams, graphs, or simulations; interpreting mathematical solutions in the context of real-life challenges; using the concept of statistical variation to make a decision; identifying mathematical aspects of a real-world problem; identifying constraints and assumptions behind mathematical modeling; representing a situation mathematically using variables, symbols, or diagrams; evaluating the significance of observed patterns in data, coding, and programming computers; working with computer mathematics systems; and calculating the properties of an irregularly shaped object.

Subjective familiarity with mathematics concepts (FAMCON) has a positive influence on math scores, β = 0.144. Mathematical terms include divisor, area of a circle, congruent figures, linear equation, and Pythagorean theorem.

However, cognitive activation in mathematics: Encourage mathematical thinking (COGACMCO) can lead to lower math performance, β = −0.095. Mathematical thinking includes teachers asking students to think of problems from everyday life that could be solved with new mathematical knowledge, showing how mathematics can be useful in our everyday lives, giving problems from everyday life involving numbers, asking students to make a decision about the situation, explaining how different mathematical ideas connect to a larger context, and so on.

Overexposure to formal and applied mathematics tasks (EXPOFA) reduces math performance, β = −0.027. Excessive and over-frequent exposure to these mathematical problems can actually be detrimental to mathematics learning.

Mathematical self-perception: Argumentation and twenty-first-century math tasks reduce math performance, β = −0.034. When students are too confident in math tasks, such as extracting mathematical information from diagrams, graphs, or simulations; interpreting mathematical solutions in the context of a real-life challenge; using the concept of statistical variation to make a decision; and so on, they may get lower math grades.

Mathematics anxiety (ANXMAT) leads to lower scores, β = −0.078. Students who often worry that mathematics classes will be difficult, get very tense when they have to do mathematics homework, feel nervous and helpless doing mathematics problems, and feel anxious about failing in mathematics usually have lower math grades.

The mathematics teaching and learning model confirms the positive effects of cognitive activation (e.g., promoting reasoning) through existing research. Balanced development of conceptual and procedural skills is essential for sustaining high-achievement trajectories (Kilp-Kabel and Mädamürk, 2024). However, excessive exposure to formalized math tasks shows negative effects, likely due to overdependence on structured practice, and a narrow mathematical perspective that limits deep understanding (Tulagan et al., 2025). Merely changing educational beliefs without altering actual teaching practices may fail to produce lasting improvements in student achievement (MacDonald et al., 2024).

In summary, exposure to and training in twenty-first-century mathematical tasks, as well as a high level of confidence in applied mathematical tasks, are conducive to enhancing students' mathematical performance. In contrast, overconfidence in mathematical tasks and overintensive training reduce mathematical performance. This can be explained by the differences in the training of basic application of mathematics and problem-solving skills, as well as the cultivation of in-depth mathematical competence and general skills; students who are skilled in basic problem-solving skills feel confident in the training of applied mathematical tasks while achieving higher mathematical performance through continuous training of general mathematical competence. On the other hand, students who fall into the training of exam-oriented education and who are overconfident and lack corresponding skills training face difficulties in mathematics learning.

*R*^2^ is the core metric for assessing the model goodness-of-fit in regression analysis, representing the proportion of variance (fluctuation) in the dependent variable (Y), which can be explained by the independent variables (X). Its value ranges from 0 to 1, with values closer to 1 indicating stronger explanatory power of the model. Overall, Model 5, incorporating math teaching and learning-related variables, demonstrated the best goodness-of-fit, with *R*^2^ = 0.511 and Δ*R*^2^ = 0.172. This suggests that math teaching and learning-related variables serve as influential factors affecting students' mathematics achievement (Table 4).

**Table 4:** Multiple regression analysis results (*n* = 6,386).

**Multiple linear regression coefficients**					
	**Model 1 Home context**	**Model 2 Information resources**	**Model 3 Personal feature**	**Model 4 School environment**	**Model 5 Math teaching and learning**
FAMSUP	0.033^**^	0.023	−0.019	−0.041^**^	−0.036^**^
FAMSUPSL	−0.184^**^	−0.173^**^	−0.173^**^	−0.195^**^	−0.128^**^
ICTRES	−0.209^**^	−0.199^**^	−0.160^**^	−0.158^**^	−0.118^**^
HOMEPOS	0.304^**^	0.285^**^	0.237^**^	0.228^**^	0.169^**^
ESCS	0.266^**^	0.243^**^	0.212^**^	0.204^**^	0.134^**^
ICTSCH		−0.004	−0.003	−0.003	0.007
ICTQUAL		0.167^**^	0.104^**^	0.060^**^	0.032^**^
ICTSUBJ		0.067^**^	0.066^**^	0.055^**^	0.057^**^
ICTENQ		0.002	−0.006	−0.020	−0.023^**^
ICTFEED		−0.116^**^	−0.123^**^	−0.138^**^	−0.132^**^
BELONG			−0.035^**^	−0.050^**^	−0.063^**^
BULLIED			−0.074^**^	−0.054^**^	−0.024^**^
FEELSAFE			0.113^**^	0.091^**^	0.056^**^
PERSEVAGR			0.094^**^	0.061^**^	−0.003
CURIOAGR			0.172^**^	0.152^**^	0.092^**^
COOPAGR			−0.078^**^	−0.081^**^	−0.044^**^
EMPATAGR			−0.021^**^	−0.028^**^	−0.025^**^
ASSERAGR			0.032^**^	0.034^**^	0.007
STRESAGR			−0.009	−0.006	−0.059^**^
EMOCOAGR			0.074^**^	0.066^**^	0.043^**^
RELATST				0.083^**^	0.054^**^
SCHSUST				0.095^**^	0.061^**^
LEARRES				0.028^**^	0.008
PROBSELF				−0.049^**^	−0.014
FEELLAH				0.018	0.020
SDLEFF				0.071^**^	0.008
DISCLIM					0.070^**^
TEACHSUP					0.025^**^
COGACRCO					0.022^**^
COGACMCO					−0.095^**^
EXPOFA					−0.027^**^
EXPO21ST					0.025^**^
MATHEFF					0.376^**^
MATHEF21					−0.034^**^
FAMCON					0.144^**^
ANXMAT					−0.078^**^
MATHPERS					0.020
Sample size	6386	6386	6386	6386	6386
*R* ^2^	0.199	0.238	0.314	0.340	0.511
*AdjustedR* ^2^	0.199	0.236	0.311	0.337	0.509
Δ*R*^2^	0.199	0.038	0.076	0.026	0.172

Dependent variable:CompScore_6378.

^*^p < 0.05 ^**^p < 0.01. The value of t is in parentheses.

## 5 Discussion

Family educational capital situation: Families with a higher socioeconomic and cultural status can provide more educational materials for students. Higher ecological sociocultural status (ESCS) was associated with higher math proficiency (Thien, 2016). Higher parental educational attainment helps obtain more home educational resources that can influence students' learning competence through real-life materials such as books and cultural capital (parental qualifications; Chang, 2023). Appropriate parental inquiry and encouragement can ease students' tension and learning anxiety, which is conducive to improving mathematics achievement (Cohen and Rubinsten, 2017). Inappropriate home study tutoring and the excessive use of ICT distracted students from learning mathematics and hindered mathematics learning.

In Model 2, with proper usage and ICT views, there was a positive correlation between properly planned use of ICT and mathematics achievement. Using digital devices as an aid to mathematics learning, students' self-confidence and scientific patterns of use were developed. The integration of ICT into the classroom increases students' motivation and learning efficiency and promotes math achievement, whereas continued engagement with ICT leads to a decrease in math achievement (Petko et al., 2017).

In Model 3, students who are able to handle emotions well, manage anxiety, and remain calm achieve higher math scores, which is caused by reducing anxiety effects on working memory, visuospatial, and knowledge capacity (Camacho-Morles et al., 2021; Živković et al., 2022). A calm mind and stable emotions are more conducive for students to think about mathematical topics, delve into mathematical research, and perform better both in class and during exams (Putwain et al., 2021; Putwain and Wood, 2022; Živković et al., 2023).

Students who study harder, are more inclusive, and attribute failures to external factors are more likely to reinforce intrinsic motivation and improve mathematics performance. Non-cognitive characteristics, such as a decrease in mathematics anxiety and improvement in self-efficacy, self-concept, and openness to problem-solving, promote mathematics achievement. Students with good learning habits and characteristics, such as conscientiousness, carefulness, curiosity, and willingness to explore, may not get higher grades, which is in contrast to previous findings (Lazarevic and Orlic, 2018).

In Model 4, teacher social-emotional support improves teacher-student relationships and provides assistance in math learning and knowledge acquisition (Wong et al., 2019). Good peer relationships are accompanied by greater empathy, which promotes math achievement as students age (Ghazy et al., 2019). In Model 4, teachers with high enthusiasm implement high-quality teaching practices, create a positive learning environment, and consider students' interests and ideas in teaching, which improves instruction effectiveness during regular classroom time and increases student self-efficacy. Students' self-efficacy improves their attitudes toward mathematics learning, increases their motivation, reduces mathematics anxiety, and promotes engagement in the mathematics classroom, which is conducive to the achievement of mathematics (Jung et al., 2023). Students with positive mathematics learning experiences tend to achieve higher mathematics achievement by accumulating mathematical knowledge, learning experiences, and cultivating mathematical abilities. Teachers' proper and scientific guidance for teaching mathematics, such as inspiring an innovative spirit, encouraging discussion, guiding thinking, and enhancing continuous exploration, is conducive to mathematics achievement (Sebastian et al., 2017). Teachers' higher-level teaching proficiency and all-around support for math teaching and learning create a positive learning environment. Teachers use scientific teaching methods and diverse teaching materials and tailor math instruction (Flannery et al., 2023).

In Model 4, students develop stronger learning competencies through higher cultural capital acquisition. This increased competence optimizes students' attitudes toward mathematics learning, enhances their positive mindset, and mediates the process of convincing them that mathematics is easy and fun to learn, thus increasing their enthusiasm for learning and mathematics achievement (Niehues et al., 2020).

Disadvantaged students who receive excessive additional academic support and too much lecturing experience student boredom and poorer achievement. Therefore, intensive training might be counterproductive. Math education requires more than simple, high-intensity teaching. This also requires proper guidance and student comprehension. Being overactive in the classroom, being too immersed in group discussions and questioning, and simply completing homework lead to poor math performance (Brezavšček et al., 2020). An increase in math study hours alone did not have a significant impact. Better math performance requires overall and comprehensive optimization and upgrading of learning time, content, mode, and practice.

In Model 5, the experience of completing mathematical tasks at school led to more exploratory behavior, potentially higher mathematics achievement scores, and a tendency to interact with the test platform more frequently when answering mathematics items (Costa and Chen, 2023). Teaching creative and typical math examples promotes the unification of students' mathematical theories and applications (Karakus et al., 2022). The scientific and proficient use of mathematical skills improves mathematics achievements. Adding knowledge about mathematical concepts and mathematical thinking to teaching content and materials benefits mathematics achievement (Braham and Libertus, 2018).

## 6 Conclusion

In Australia, the main factors influencing PISA math performance are family support, the process of learning math, and individual student characteristics.

Home context, such as support from the family's socioeconomic status, educational capital, and parents' educational background, had a positive influence. Excessive use of ICT, on the other hand, has a negative impact on math achievement. Students' high sense of security, resilience, curiosity, strong will, and good emotional management were conducive to mathematics achievement.

Good teacher-student relationships and sustainable learning actions increase mathematics achievements. High-quality teaching and learning processes of teachers, a favorable mathematical climate, and students' correct attitudes toward learning mathematics are key. Blindly increasing the mathematics curriculum to train students too much in the subject and the over-activation and immersion of students in classroom discussion and questioning may be counterproductive. In teaching, mathematical reasoning and twenty-first-century mathematics tasks contribute to students' math performance. Mathematics teaching should focus on mathematical cognitive activation, enhance students' mathematical self-awareness, and emphasize mathematical conceptual guidance. An effective teaching and learning process can prevent students from spending too much energy in the wrong direction or focusing too much on side issues. Mathematics teaching content is well-designed, application-oriented teaching tasks have been developed, and teacher guidance can maximize its positive effects.

## 7 Research value, limitations, and future research

In terms of limitations, the data were solely drawn from the PISA 2022 dataset, and while the sample size of 6,386 is adequate, it may not fully represent the entire Australian student population. Methodologically, the use of multiple regression analysis instead of hierarchical linear modeling (HLM), although theoretically justified, may insufficiently capture nested effects at the regional, school, and classroom levels. Although the model fit indices met acceptable thresholds, they fell short of the optimal standards, indicating room for improvement. Additionally, the study's reliance on cross-sectional data precludes causal inferences among variables and lacks longitudinal time-series tracking investigations.

In terms of implications for future research, future research could employ longitudinal designs to track changes in students' mathematics achievement, thereby establishing causal relationships among various factors. Qualitative methods (e.g., interviews and classroom observations) could be incorporated to provide deeper insights into the specific mechanisms underlying mathematics teaching and learning processes, particularly how EXPO21ST enhances achievement. The scope could be expanded to compare Australia's mathematics education system with that of other Western countries. More sophisticated statistical modeling (e.g., structural equation modeling) could be adopted to analyze cross-level interactions among factors, with special attention paid to the interplay between family background and school environments.

In terms of theoretical contribution, this study innovatively applies Bronfenbrenner's ecological systems theory to mathematics education research by constructing a dual-level nested ecosystem model that specifically identifies mathematics teaching and learning as a key dimension. In terms of practical contributions, the study confirms the significant impact of family support and economic-cultural status (ESC) on mathematics achievement while highlighting the crucial role of mathematics self-efficacy (MATHEFF), providing empirical evidence for educational policymaking. The research also identified the positive effects of ICTQUAL (ICT quality), TEACHSUP (teacher support), and EXPO21ST (exposure to twenty-first-century tasks), offering concrete directions for classroom instructional design and underscoring the importance of mathematics teacher development.

This study makes significant contributions by addressing a research gap in empirical studies of Australia's mathematics education system, particularly in analyzing factors influencing its upward trend in PISA mathematics achievement. This study innovatively incorporated mathematics teaching content and process variables into the analytical framework, revealing the crucial roles of mathematics self-efficacy (MATHEFF), conceptual familiarity, and instructional task design, thereby expanding existing research dimensions. These findings provide actionable recommendations for Australia and similar education systems, such as optimizing family support strategies, balancing ICT use, and enhancing mathematical reasoning task training. This methodological approach offers a replicable analytical framework for similar educational assessment studies.

## Data Availability

The original contributions presented in the study are included in the article/supplementary material, further inquiries can be directed to the corresponding author.
